# Investigations of Physical Compatibilities of Commonly Used Intravenous Medications with and without Parenteral Nutrition in Pediatric Cardiovascular Intensive Care Unit Patients

**DOI:** 10.3390/ph12020067

**Published:** 2019-05-04

**Authors:** Katherine Greenhill, Erin Hornsby, Greg Gorman

**Affiliations:** McWhorter School of Pharmacy, Samford University, Birmingham city, AL 35229, USA; kgreenhi@samford.edu (K.G.); ehornsby@samford.edu (E.H.)

**Keywords:** Y-site, drug compatibility, pediatric, cardiovascular

## Abstract

Many pediatric intensive care patients require numerous specialized intravenous (IV) medications at various dosages in multiple fluids often with nutritional support. This requires several venous access points due to lack of Y-site compatibility data for combinations of two or more drugs. This project investigated physical compatibilities of intravenous medications: alprostadil, calcium gluconate, dexmedetomidine, epinephrine, norepinephrine, esmolol, furosemide, vasopressin, and milrinone with and without lipid-free total parenteral nutrition (TPN) commonly used in a pediatric cardiovascular intensive care unit (CVICU) patient. Actual drug combinations were evaluated using a simulated Y-site study design. Compatibility was determined based on observational data: odor (change/appearance), evolution of gas, and visual appearance combined with physical or chemical endpoints with predefined acceptance criteria: change in pH (± 1 unit), and turbidity (>0.5 NTU) at eight time points between 0 and 240 min. All binary drug combinations along with the four drug plus TPN combination were found to be physically compatible up to 240 min. The three drug combinations were determined to be incompatible and were not evaluated with TPN. This study demonstrates the utility of simulated Y-site study design to multi-drug combinations and increases the scientific body of knowledge related to medications used in a pediatric CVICU.

## 1. Introduction

Pediatric patients (neonates through 18 years of age) placed in an intensive care unit (ICU) require multiple intravenous medications and/or parenteral nutrition. Typically these medications may be administered together through a Y-site connection; however, depending on physical compatibilities, patients may also require multiple access points, which can be difficult to establish [1,2]. In addition to finding other venous access points, presence of additional entry locations may increase the risk of complications related to infection or thromboembolism [2]. For patients with continuous infusions, temporarily stopping medications or nutrition may be necessary in order to administer other medications that are incompatible or with unknown compatibilities. Patients requiring continuous total parenteral nutrition (TPN) have greater complexity in their regimen due to the multiple components within TPN formulations which could potentially interact with medications. This may lead to clinically suboptimal outcomes, or in the case of parenteral nutrition, malnutrition [1]. Alternatively, studies have shown that when compatibility data is missing, medications are likely to be mixed together inappropriately [2]. This can lead to events such as pulmonary embolism in the case of TPNs containing calcium and phosphorus when mixed with medications that can induce precipitate formation [1].

Pediatric patients present further challenges when administering medications via IV route. Information regarding physical compatibility may not be as readily available as it is for adults due to differences in doses, vehicles, and parenteral nutrition requirements. Furthermore, available information that does exist cannot simply be extrapolated to pediatric patients [3,4,5]. Due to body composition, their veins are harder to visualize and they are more likely to become distressed and uncooperative during venipuncture [6]. Additionally, repeated flushing of IV lines prior to and after drug administration may pose problematic for pediatric patients in relation to hypervolemia due to small fluid capacity. This would require that medications and TPN use the same line for infusion [1,4].

A device that may be utilized to overcome the issue of limited access sites is known as a multiport manifold device. This device follows the same concept as a Y-site connector but can instead administer up to seven different medications through one IV line. A potential issue with the use of a multiport device is that infusing numerous medications, with or without TPN, into one central line at slow infusion rates could lead to development of drug incompatibilities. With a deficit in compatibility data available regarding the administration of multiple drugs through the same central venous line using a Y-site set or multiport manifold, healthcare providers are more reluctant to co-administer multiple medications in this manner [3].

In a previous study from a single center, researchers assessed the physical compatibility of a lipid-free TPN in combination with a standard mixture of epinephrine, vasopressin, milrinone, and calcium gluconate with the following: furosemide, esmolol, amiodarone, and dexmedetomidine. Results from this study demonstrated that the standard mixture with the lipid-free TPN was compatible with all the previously listed medications except for amiodarone [7]. This study seeks to build upon these results and expand clinical utility to additional medications and combinations. The objective of this study was to investigate the physical compatibilities of intravenous medications in circumstances where data for institutional specific pediatric concentrations and/or vehicles does not exist, or for multiple medications in the presence of a common TPN formulation in pediatric cardiovascular intensive care unit (CVICU) patients.

## 2. Methods and Materials

### 2.1. Materials

The formulated medications were provided by the pharmacy staff at the partnered institution (Children’s of Alabama) with the exception of furosemide and a limited quantity of dextrose 5% in water (D5W) vehicle which were prepared in our laboratory. Due to the large volume required for the experiments, furosemide was prepared in the lab each day of the experiments using the drug stock powder dissolved in normal saline with pH correction using sodium hydroxide solution and mild heat to allow dissolution into the vehicle. D5W was obtained from the partnered institution for Phase I of the experiments, but made in the lab for the second and third phases due to limited commercial availability. The concentrations chosen were based on dialog with the clinicians at the partnered institution for clinically relevant doses and combinations used in their pediatric CVICU. The manufacturer, lot numbers, expiration dates, and stock concentrations of the drugs, materials, and vehicles used for this project are listed in Table 1. Formulation details of the selected lipid-free TPN solution are listed in Table 2. All medications were used prior to their expiration dates.

### 2.2. Design

This study was conducted in three separate phases. Phase I assessed physical compatibility of various binary drug combinations prepared in a 1:1 ratio in different vehicles. Phase II evaluated furosemide and milrinone at pediatric relevant dosages (10 and 1 mg/mL, respectively) with up to three additional medications to determine if dilution effects of vehicle from the additional medications could potentially render the mixtures compatible. It should be noted that furosemide and milrinone together have previously been reported as incompatible at 10 and 0.2 mg/mL, respectively [8,9]. In phase III, a lipid-free TPN formulation was evaluated with the four most commonly administered medications in the institutional unit at clinically relevant concentrations. The TPN, being lipid-free, allowed for visual and turbidimetric evaluations and should be noted as containing the maximum amounts of calcium and phosphorus for this patient population. The evaluated drug combinations for all three phases at respective concentrations and vehicles compositions are listed in Table 3, Table 4 and Table 5. A total volume of 100 mL of each combination was prepared by sequentially mixing together the medications in equal volumes in glass beakers at their clinically dosed concentrations in the same order for each iteration.

All drugs and TPN were stored according to the manufacturers’ recommendations prior to being used in the study. The use of equal volumes of drugs with TPN in simulated Y-site compatibility studies have previously been verified as a standard practice [10].

### 2.3. Data Collection

Each drug combination was evaluated for pH, turbidity, evolution of gas, color, and formation of precipitate upon mixing at time = 0 (baseline), and additionally at 5, 15, 30, 45, 60, 120, and 240 min by two independent evaluators. The evaluation time frame was based on common drip rates used to determine actual time that the medications would be in contact in an IV line prior to entering a patient, with the maximum time point also being used in other studies [1,3,10,11]. The presence of two evaluators also aided in the reduction of potential bias in visual assessments [2]. Measurements at each time point were compared to baseline values. Multiple methods were chosen to assess for physical incompatibility due to the complexity of each drug mixture as recommended in published studies [1]. The pH (Ohaus Starter pH meter, Parsippany, NJ) was assessed with changes of ± 1.0 pH unit or more from baseline deemed physically incompatible. Changes in pH can contribute to precipitation as well as suggest the presence of chemical reactions [2,3]. Turbidity (2100Q Turbidimeter, Hach, Loveland, CO, USA) was assessed with changes of < 0.5 nephelometric turbidity units (NTU) deemed as compatible. This standard has been reported in other studies to be an appropriate threshold in which to demonstrate physical compatibility [11]. Both the pH meter and the turbidimeter were calibrated each day prior to testing as described in the manufacturers’ instruction manual. Gas formation was noted if there was bubble formation upon mixing or during any of the observation time points. For visual assessments, two evaluators observed the solutions in front of non-glossy black, white, and no background to assess for formation of precipitate and color change. All visual assessments were performed under LED laboratory lighting.

## 3. Results

**Figure 1:**
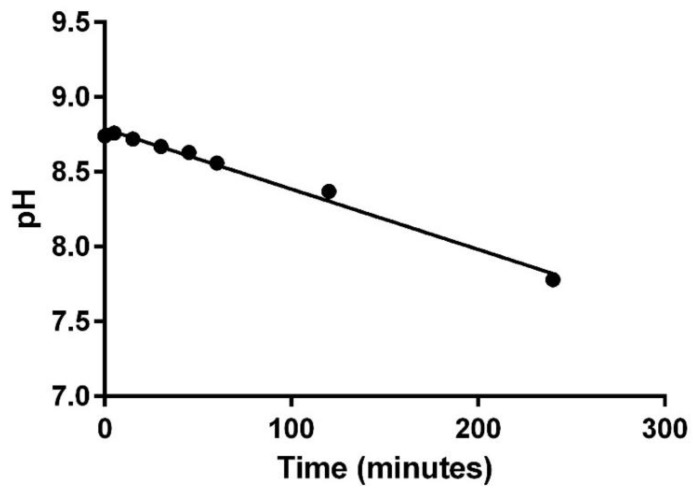
pH change for furosemide (1 mg/mL) with epinephrine (16 mcg/mL).

The overall physical compatibility results of this study for each combination at 240 min are shown in Table 3, Table 4 and Table 5. The observed pH range, turbidity range, visual appearance, odor, and gas evolution values from each combination from all phases of this study are reported in Table 6. Reported changes in turbidity and pH were calculated by subtracting the lowest or highest measured value from the baseline measurement, whichever value represented the largest difference. Of the thirteen combinations assessed in phase I, all were found to be physically compatible up to the maximum 240 min based on the previously defined assessment criteria. The combination of furosemide (1 mg/mL) with epinephrine (16 mcg/mL) was closest to exceeding the threshold for change in pH at the 240-minute time point, as it began trending down after the first measured time point (Figure 1). Continued evaluations beyond the 240-minute time point would have resulted in a pH change of greater than 1 making this combination incompatible. Additionally, the combination of furosemide (1 mg/mL) with alprostadil (10 mcg/mL) displayed a slight positive trending slope in measured turbidity which could have potentially exceeded 1 NTU but not until some point well beyond 240 min. All other combinations had minimal non-trending changes in the measured parameters. Phase II multi-drug combinations containing three or more medications were all found to be physically incompatible (Table 4). Each combination in this phase immediately produced a milky-white, fluent, and cloud-like precipitate, similar to the effect of food-coloring, when milrinone (3rd drug in sequence) was added; however, this fluent precipitate quickly dissipated within a few seconds after milrinone was completely added. Combinations II.1 and II.2 also formed white speck-like precipitates within five minutes of all drugs being added to the mixture and combination II.3 formed a milky-white precipitate similar in appearance to the initial precipitate immediately after mixing of all five drugs. While all three combinations were determined incompatible due to turbidity changes of ≥ 0.5 NTU, combinations II.1 and II. 2 were still below the physical incompatibility threshold for pH despite the drastic increases in turbidity and precipitate formation that was observed. The pH was only tested at baseline for II.3 because of the immediate and stable precipitate formation that occurred upon mixing of all five drugs. No changes in odor or observation of evolved gases were noted in any of the phase II drug combinations. In phase III the four drug combination evaluated with TPN was found to be physically compatible through the 240-minute observation period in all parameters measured. As with the phase II combinations, no changes in odor or observation of evolved gases were noted. Furthermore, measured changes in both pH and turbidity for the phase III combination were very minimal with no observed trends.

## 4. Discussion

Critically ill patients placed in intensive care units typically require multiple IV medications, while others may also require parenteral nutrition. Unknown or known physical incompatibilities would require that medications be given through separate IV lines. In cases of limited or no additional venous access, infused medications or TPN may have to be temporarily discontinued in order to administer other medications, requiring multiple saline flushes throughout the day. For pediatric patients, establishing extra IV access may be difficult due to their level of agitation, physical mobility, and body composition. Using additional flushes to administer medications or nutrition through the same IV line could be detrimental to the patient depending on volume status. Additionally, information regarding physical compatibilities of medications at pediatric specific doses is not as extensive as it is for adults. Extrapolation of information from adult to pediatric patients is not feasible due to the variations in nutrition requirements and concentrations needed for medications due to volume issues and dosing. This gap in published data demonstrates there is a need for physical compatibility data for medications commonly used in pediatric patients.

This study helps to bridge this paucity of data by yielding results from multiple combinations of medications with a complex TPN solution. The data collected from this study shows that the majority of the routinely used clinical combinations evaluated were able to demonstrate physical compatibility. The investigators recognize that the results of this study may not be applicable to every institution or patient circumstance due to the design being specific to the doses and combinations used at the partnered institution.

Overall, the binary combinations of phase I were all found to be physically compatible based on our predefined physical incompatibility evaluation criteria. However, two of the combinations were observed to have trending data suggesting imminent incompatibility. Overall, due to the trend in pH and turbidity in combinations I.5 and I.10, respectively, caution should be taken when using these combinations at the given concentrations. According to the IV compatibility data in Lexicomp and Micromedex, which are both powered by Trissel’s, alprostadil and furosemide are compatible for IV Y-site administration [8,9]. The study compared a lower concentration of alprostadil in a different vehicle (7.5 mcg/mL in D5W) with a higher concentration of furosemide (5 mg/mL) in an unknown vehicle over 60 min using only visual observations and evolution of gas as endpoints. With the absence of trending pH and turbidity data in this study the potential for incompatibility could not be predicted [12].

Previous data published in Trissel’s for esmolol and furosemide show they are incompatible; however, these studies were conducted at different concentrations and with different vehicles as compared to our study. Esmolol concentrations were either at 10 mg/mL or 40 mg/mL in either D5W, NS, or undiluted. The esmolol concentration in this study of 20 mg/mL falls in between; however, the furosemide concentration used in this study was much lower than reported in Trissel’s, 1 mg/mL vs. 5–10 mg/mL, respectively. Additionally, the furosemide reported in Trissel’s was either undiluted or dissolved in D5W solvent as opposed to NS in this study. The only study reported in Trissel’s that used furosemide in NS compared the 5 mg/mL to esmolol 40 mg/mL When compared to this study, the concentration of esmolol and furosemide were twice and five times as high, respectively [8,9]. These large differences in concentrations between the study reported in Trissel’s and this study further show the void in drug compatibility data for pediatric dosages and highlight the importance of drug compatibility studies targeting common doses and concentrations for pediatric patients.

In the case of epinephrine and furosemide, Trissel’s shows that they are compatible via Y-site connection; however, the concentrations and vehicles used were again different than the ones used in this study. For norepinephrine and furosemide, Trissel’s shows they are compatible when norepinephrine is at a lower concentrations (128 mcg/mL and below) and incompatible when norepinephrine is at 0.5 mg/mL [8,9]. This study used lower concentrations that would be appropriate for pediatric patients, which is likely why the two were physically compatible.

Per Micromedex and Lexicomp (Trissel’s), the Y-site compatibility of furosemide and norepinephrine bitartrate is reported as variable primarily due to a function of concentration [8,9]. At norepinephrine concentrations greater than 128 mcg/mL combined with furosemide at 5 mg/mL in all fluids tested, the combinations were found to be incompatible. For concentrations of norepinephrine at or below 128 mcg/mL with furosemide as high as 10 mg/mL the combinations are reported as compatible. Of the 4 pediatric dose combinations of furosemide and norepinephrine we evaluated, all were found to be compatible (Table 3); however, only one of the concentration pairs (furosemide 10 mg/mL with norepinephrine 100 mcg/mL) could be found in the database. Even still, the vehicle was different from that used in this study.

The y-site compatibility data resulting from nine listings comparing furosemide and vasopressin have variable results, according to Micromedex and Lexicomp [8,9]. One study used the same concentrations of each medication as we report in this study with the same results but in a different vehicle and for 24 hours [13]. The remaining studies evaluated furosemide at either 4 or 5 mg/mL with vasopressin at either 0.4 or 4 units/mL. Interestingly, incompatibility occurred when vasopressin and furosemide were both at the lower concentration (0.4 units/mL and 4 mg/mL, respectively) regardless of the vehicle [14].

All combinations of Phase II failed physical compatibility based on the predefined evaluation criteria with the formation of precipitates and an increase in turbidity measurements of ≥ 0.5 NTU (Table 4). Milrinone (200 mcg/mL) in D5W and furosemide (10 mg/mL) are known to be incompatible drugs for Y-site administration, per Lexicomp and Micromedex [8,9]. In this study, it was found that upon mixing milrinone (1000 mcg/mL) in NS with furosemide (10 mg/mL) in NS, precipitation occurred regardless of the addition of more drugs in attempt to further dilute furosemide and milrinone. According to Micromedex and Lexicomp, the only drugs of the 5-drug combination that are not compatible are: (1) furosemide and milrinone and (2) furosemide and vasopressin, which had variable data; however, from Phase I data we found that the combinations of furosemide (10 mg/mL and 1 mg/mL) in NS with vasopressin (1 unit/mL) in NS were physically compatible [8,9]. Thus, it can be concluded that the precipitation formed during the testing of drugs in phase II must be due to the incompatibilities of furosemide and milrinone. The order in which the medications were mixed could have affected the outcome of our study, but the addition order was chosen so that milrinone would be added last for combination II.1, the first combination that was tested. In addition to this, the order in which the medications were added was kept synonymous for combinations II.2 and II.3, so milrinone was added third to the mixtures and not added last for II.2 and II.3. Even if the order in which milrinone was added in phase II was changed and subsequently produced a positive outcome, it would still be unlikely to affect the use of this combination in clinical practice since the order in which the medications enter the Y-site multiport would realistically be much harder to control.

The results from phase III of epinephrine 100 mcg/mL, milrinone 1000 mcg/mL, vasopressin 1 unit/mL, and calcium gluconate 100 mg/mL with lipid-free TPN showed the combination to be compatible. The use of this combination has since been successfully implemented in clinical practice by the partnered institution. As a result, calcium and phosphorus are no longer left out of parenteral nutrition for patients requiring calcium gluconate infusions. Therefore, this has the potential to reduce the incidence and risk of hypophosphatemia for patients requiring calcium gluconate infusions for longer than 48 hours which could require IV phosphate supplementation. An important consideration regarding this study is the TPN solution used was lipid-free. Compared to lipid containing TPN solutions which are opaque and non-transparent emulsions, lipid-free TPN, while yellow in color, is visually clear and allows for visual and turbidimetric evaluation of the drug mixture to assess chemical compatibility. These assessment endpoints could not be used in drug mixtures with lipid containing TPN solutions, thus making it difficult to determine physical compatibility. The information gathered from this study may make it more feasible to use a multiport manifold device for patients requiring several IV medications, reducing the need for multiple IV lines.

## 5. Conclusions

In conclusion, all combinations assessed in Phase I and III of this project proved to be physically compatible up to 240 min, while the combinations from Phase II were all incompatible. The results from this study significantly add to the body of knowledge in the field of drug Y-site compatibility for dosages and vehicles used for pediatric patients. Clinicians should be cautious when extrapolating the data, noting that the TPN used did not contain lipids and the concentrations of the TPN components and drugs used may not be applicable to every patient. Additionally, using vehicles different from the ones used in this study may impact the physical compatibility to yield different results than were produced here.

## Figures and Tables

**Table 1:** Materials and vehicles.

Drug/Vehicle	Supplied Concentration	Manufacturer	Lot Number	Expiration Date
Alprostadil	500 mcg/mL	Pharmacia and Upjohn Co.	R75137	06/2019
Calcium gluconate	1 mg/mL	Fresenius Kabi	6013761	04/2018
Dexmedetomidine HCl	400 mcg/mL	Hospira	74160DD	02/2019
Dexmedetomidine	200 mcg/2 mL	Intas Pharm. Limited	W08976	05/2019
Epinephrine HCl	1 mg/mL	Amphastar	DT020C7	02/2019
Esmolol	20 mg/mL	Baxter	Y225839	02/2019
Furosemide	Bulk	Letco	1502110214	02/2018
Milrinone Lactate Inj	1 mg/mL	Hikma West-Ward Pharmaceutical	1510491	03/2018
Milrinone Lactate Inj	1 mg/mL	APP Pharmaceuticals	6008428	08/2017
Norepinephrine Bitartrate	1 mg/mL	Hospira	740653A	08/2018
Vasopressin	20 units/mL	Par Pharmaceutical	818725	08/2018
5% Dextrose in water	5%	Baxter	P352880	02/2018
Dextrose Anhydrous	Not Applicable	Letco	1601050027	02/6/18
Normal Saline	0.9%	Baxter	Y230961	10/2018
Sodium Hydroxide	97% flakes	Letco	1601050027	02/2018

**Table 2:** Total parenteral nutrition (TPN) composition.

Ingredient	Concentration
Dextrose	25%
Travasol ^1^	3%
Sodium	150 mEq/L
Potassium	80 mEq/L
Magnesium	5 mEq/L
Calcium	18 mEq/L
Chloride	75 mEq/L
Phosphorus	7 mmol/L
Acetate	75 mEq/L
Infuvite Pediatric Multivitamin ^2^	5 mL
Selenium	10 mcg/L
Multitrace-4 Concentrate ^3^	1 mL
Heparin	1000 units/L

^1^https://www.baxter.ca/sites/g/files/ebysai1431/files/2018-12/8_Travasol_E_Travasol_EN.pdf; ^2^http://www.baxtermedicationdeliveryproducts.com/pdf/VitaminsPediatricPI.pdf; ^3^https://www.americanregent.com/media/2211/multitrace-4-sds-03jan2019.pdf.

**Table 3:** Phase I overall physical compatibility results.

Phase I Combinations		
Drug	Furosemide 1 mg/mL	Furosemide 10 mg/mL
Epinephrine 16 mcg/mL	Compatible *	Compatible
Epinephrine 100 mcg/mL	Compatible	Compatible
Norepinephrine 16 mcg/mL	Compatible	Compatible
Norepinephrine 100 mcg/mL **	Compatible	Compatible
Vasopressin 1 unit/mL	Compatible	Compatible
Esmolol 20 mg/mL	Compatible	Not Tested
Alprostadil 10 mcg/mL	Compatible	Not Tested
Dexmedetomidine 4 mcg/mL	Not Tested	Compatible

* This combination exhibited a negative trending pH slope which would have exceeded the compatibility criteria at measurements beyond 240 min. ** Denotes drug was in 5% Dextrose in water. All other drugs were in normal saline.

**Table 4:** Phase II.1-II.3 overall physical compatibility results.

Phase II Combinations *					
Drug 1	Drug 2	Drug 3	Drug 4	Drug 5	Results
Furosemide 10 mg/mL	Epinephrine 100 mcg/mL	Milrinone 1000 mcg/mL	NA	NA	Incompatible
Furosemide 10 mg/mL	Epinephrine 100 mcg/mL	Milrinone 1000 mcg/mL	Dexmedetomidine 4 mcg/mL	NA	Incompatible
Furosemide 10 mg/mL	Epinephrine 100 mcg/mL	Milrinone 1000 mcg/mL	Dexmedetomidine 4 mcg/mL	Vasopressin 1 unit/mL	Incompatible

* Drugs listed in order of addition from left to right. All drugs in normal saline except vasopressin (D5W).

**Table 5:** Phase III overall physical compatibility results.

Phase III Combination					
Nutrition	Drug 1 concentration, vehicle	Drug 2 concentration, vehicle	Drug 3 concentration, vehicle	Drug 4 concentration, vehicle	Result
Lipid-free TPN *	Epinephrine 100 mcg/mL, NS	Milrinone 1000 mcg/mL	Vasopressin 1 unit/mL, D5W	Calcium gluconate 100 mg/mL	Compatible

* see Table 2 for composition of lipid-free TPN.

**Table 6:** Results of the physical compatibility of various solutions at 240 min.

Combination	Visual Changes	Turbidity Changes (NTU)	pH Changes (pH Units)	Odor	Evolution of Gas
**Phase I**					
1	None	0.06 (0.48–0.54)	−0.06 (4.90–4.96)	None	None
2	None	0.14 (0.97–1.11)	0.31 (9.19–9.50)	None	None
3	None	0.24 (0.55–0.79)	−0.10 (6.41–6.51)	None	None
4	Clear, light yellow	0.21 (0.9–1.11)	0.28 (9.55–9.83)	None	None
5	None	−0.37 (0.43–0.80)	−0.96 (7.78–8.74)	None	None
6	Clear, light yellow	0.15 (0.75–0.90)	0.11 (9.41–9.52)	None	None
7	None	−0.18 (0.34–0.52)	0.11 (5.31–5.42)	None	None
8	None	0.01 (0.87–0.88)	−0.07 (9.71–9.78)	None	None
9	None	−0.10 (0.33–0.43)	−0.43 (8.56–8.99)	None	None
10	None	0.30 (1.78–2.08)	−0.57 (8.99–9.56)	None	None
11	None	0.01 (0.93–0.94)	−0.07 (9.74–9.81)	None	None
12	None	−0.11 (0.96–1.07)	−0.08 (9.67–9.75)	None	None
13	None	0.01 (0.34–0.35)	−0.19 (6.63–6.82)	None	None
**Phase II**					
1	White precipitate formation	3.6 (3.88–7.48) ^a^	−0.09 (4.68–4.77)	None	None
2	White precipitate formation	6.14 (0.84–6.98) ^b^	0.09 (4.73–4.82)	None	None
3	White precipitate formation	22.14 (1.06–23.3) ^b^	4.67	None	None
**Phase III**					
1	None	−0.08 (0.54–0.62)	0.08 (5.55–5.63)	None	None

^a^ represents an observation period of 5 min ^b^ represents an observation period of 15 min.
